# Testing influences of openness, conscientiousness, nationalism, media diversity, social class, and informational echo chambers on support for official responses to COVID-19 in Wuhan in November, 2020

**DOI:** 10.3389/fpsyg.2024.1370870

**Published:** 2024-05-22

**Authors:** Bo Miao, Hanqing Ding

**Affiliations:** ^1^School of International Communication and Arts, Hainan University, Haikou, China; ^2^School of Journalism and Communication, Beijing Normal University, Beijing, China

**Keywords:** personality, ideology, media use, echo chamber acts, political attitude, triadic reciprocal determinism

## Abstract

**Introduction:**

As the COVID-19 pandemic raged, controversies about governmental responses to the epidemic also emerged in China. Previous studies mainly described the phenomenon of individual differences on support for governmental responses to COVID-19 with less attention to the underlying causal mechanisms. Thus, this study tries to verify the factors influencing public support for official behaviors in COVID-19.

**Method:**

A questionnaire survey was drew on in Wuhan city during the COVID-19 outbreak. The quota sampling method was adopted according to the gender and age structure of the population in Wuhan as well as the educational structure of the urban population in China.

**Results:**

Through structural equation analysis, this study confirms that personal factors (namely conscientiousness and nationalistic ideology), behavioral factors (namely media diversity and echo chamber acts) exert significantly positive impacts on support for governmental responses. The echo chamber acts play important mediating roles in the relationship between each independent variable and support for governmental responses.

**Discussion:**

The originality of this study is that it constructs a comprehensive model of influencing factors of support for governmental responses with the personal, behavioral, and environmental factors. While contributing insight to political attitude in China, the research results also have significance for promoting public trust and constructing healthy public opinion in China.

## 1 Introduction

In the end of 2019, the COVID-19 pandemic swept all the world leading to a massive health crisis and uncertainty of human society. Disputes about official behaviors coping with the epidemic also emerged in China. For example, some people thought that lockdown policy in China was an indispensable epidemic prevention measure, while others considered that strict control led to impaired individual freedom and an increase in loneliness (Völker, 2023); some people thought that the British government should learn from Chinese government and abort the “herd immunity” policy, while others argued that it was too arrogant to let other countries learn from China. Controversies about Chinese official actions have been found in many socio-political incidents (Pan and Shu, 2023). The conservative and nationalistic people always strongly support Chinese government, while the liberal public often take the opposite position. Existing studies generally found viewpoint differences and opinion polarization in China, but these studies stayed at the level of descriptive analysis and paid less attention to the underlying formation mechanisms (Ma and Huang, 2023). Hence, it is necessary to analyze the underlying causal mechanisms of public support for official behaviors in COVID-19 in China.

Most studies on political attitude have regarded one aspect of the influencing factors. For example, (1) psychological studies attached importance to personal inner factors such as personality traits, and found that open individuals were more likely to hold liberal stance (Mirels and Dean, 2006; Schoen and Schumann, 2007; Gerber et al., 2011; Onraet et al., 2011), but conscientious persons were more conservative in political attitude (Carney et al., 2008). (2) media studies emphasized the impact of media usage and confirmed that political attitude was not only determined by internal factors, but also influenced by the pseudo-environment provided by media (Tang, 2008; Willnat and Metzgar, 2012; Zhang and Xu, 2023); (3) the sociological studies mainly explored the influences of social structural factors such as social class, and found that people from different social classes have formed distinct cognitive tendencies (Kraus et al., 2012; Hu and Lin, 2022). Few studies have integrated the three study perspectives and systematically investigated the overall impact paths of three categories of factors, which might result in the neglect of some key mediating effects. Based on theory of triadic reciprocal determinism (Albert, 1986, 2001), this study extends the research scope by constructing a comprehensive model of the factors influencing public support for official behaviors in COVID-19. The model includes personal factors (namely personality and nationalistic ideology), behavioral factors (namely media diversity, which means the number of media sources in a person’s media repertoire in COVID-19, and echo chamber acts, which indicates the homogeneous information that people contact with in COVID-19), and environmental factors (namely social class).

From the perspective of media study, relevant researches about political attitude mainly classified media by its channel type (Willnat and Metzgar, 2012; Ji and Xiao, 2023). This is not suitable for the high-choice media environment generated by the Internet nowadays (Dubois and Blank, 2018). Therefore, this study attempts to measure the diversity (namely media diversity) and homogeneity (namely echo chamber acts) of media contact behaviors, instead of measuring the use of different media types. And the effects of these two variables on public support for governmental responses to COVID-19 are verified through empirical survey.

In a nutshell, this study contributes to the previous literature from three aspects: (1) a comprehensive model of factors influencing public support for official behaviors is constructed from three aspects: personal, behavioral, and environmental factors; (2) to represent the high-choice media environment, the diversity (namely media diversity) and homogeneity (namely echo chamber acts) of media contact behaviors are measured; (3) the influences of media diversity and echo chamber acts on public support for official behaviors are verified through empirical study.

The following section reviews the triadic reciprocal determinism and previous literature related to influencing factors of political attitude. A hypothesis model is proposed based on triadic reciprocal determinism and existing literature. Furthermore, the questionnaire survey method is introduced. Next, we analyze our theoretical model using structural equation model and find that echo chamber acts play important mediating roles in the theoretical model. In the end, we conclude with a discussion of the reasons why some factors influence support for governmental responses to COVID-19, and practical suggestions for government as well as Internet platforms are proposed based on our results.

## 2 Literature review and hypotheses

### 2.1 Theoretical background: triadic reciprocal determinism

Introduced by Albert Bandura, triadic reciprocal determinism theory indicated that human thoughts were not just driven by inner forces, nor only affected by external incentives, whereas the person, behavior, and environment formed a triangle relationship structure, in which two of the above entities influenced each other in a reciprocal way (Albert, 1986; Albert and Forest, 1991; Albert, 2001; Nong et al., 2022).

The adoption of triadic reciprocal determinism as the fundamental theory is appropriate for present study for two reasons. Firstly, it can serve as an overarching framework to explore why people hold different views on governmental behaviors in COVID-19, which guides us to consider the roles of personal, behavioral, and environmental factors; Secondly, the theory postulates the reciprocal influences, which leads us to examine the mediating roles of echo chamber acts.

### 2.2 Personal factors: personality and ideology

Triadic reciprocal determinism is based on the idea that people are not merely organisms that respond to external events, but also are regulated by internal factors. Reviewing the existing studies, it was found that personality and ideology were the main factors that affected public support for government.

#### 2.2.1 Personality

Personality plays a fundamental and guiding role in the perception of political incidents (Ma and Zhang, 2017). Previous studies using the Big Five Model found that openness and conscientiousness were the main factors affected public viewpoints of political issues, and the influences of openness and conscientiousness were relatively stable compared with other personality traits (Ma and Wang, 2015).

Individuals with low degree of openness are more likely to form right-wing attitude, support authoritarianism and tradition, as well as resist change and new information (Onraet et al., 2011; Ma and Zhang, 2017; Beattie et al., 2022). In contrast, individuals with high-level openness tend to adhere to the liberal stance, oppose authority such as government authority (Schoen and Schumann, 2007; Carney et al., 2008; Beattie et al., 2022; Ma and Lu, 2022). In other words, with higher level of openness, people usually are less likely to support official behaviors. Thus, we put forward H1a.

*H1a*: Openness negatively predicts public support for official behaviors in COVID-19.

For conscientious personality, it is associated with attitude of rigidity, order, and control of the environment. Individuals with high-level conscientiousness require order and are unwilling to accept uncertainties (Jost et al., 2003; Hiel et al., 2004; Kruglanski and Boyatzi, 2012), so they are more inclined to hold conservative positions, identify with authority, and resist changes (Carney et al., 2008; Gerber et al., 2011; Ma and Lu, 2022). In COVID-19, the uncertainties of social environment have greatly increased. As a result, individuals with high-level conscientiousness might prefer to restore the social order and support the control measures conducted by the Chinese government. Thus, H1b is proposed.

*H1b*: Conscientiousness positively predicts public support for official behaviors in COVID-19.

#### 2.2.2 Ideology

With limited knowledge and time, people hardly understand the whole picture of an incident. Thus, the existing ideology provides individuals with a lens through which they process information and make judgments on the political incidents (Jost et al., 2009; Ma and Wang, 2015). It was found that public standpoints of political incidents were driven primarily by ideology (Kahan, 2017; Bavel and Pereira, 2018).

For purpose of analysis we identify one ideological position that public might take: nationalism. Nationalism as concept has multiple dimensions. Smith summarized five most widely adopted meanings of the concept of nationalism: (1) the formation, or growth, of nations; (2) a consciousness of belonging to a nation; (3) a symbolism of nation; (4) a social and political movement on behalf of the nation; and (5) a doctrine and/or ideology of the nation (Smith, 2010). According to Smith’s definition, nationalism is closely associated with the concept “patriotism,” both of which emphasize the attachment and belonging to one’s country. But there are differences between the two concepts. Nationalism is a conviction of the superiority of one’s country over other countries, but patriotism does not contain a sense of superiority (Kosterman and Feshbach, 1989).

For Chinese nationalistic ideology, there was no concept of nationalism in ancient feudalist China since the country was largely ruled by Confucian ideologies (Ma Y., 2015). It was not until the late nineteenth century when China was invaded by the West and Japan when the nationalism was introduced to China. Reformers like Sun Yet-sen and Mao Zedong started to use nationalism to unite Chinese people and mobilize them to fight against both external enemies and internal feudalism (Liu and Zhou, 2019). After the founding of People’s Republic of China in 1949, the Chinese government established a “militant, revolutionary, and anti-imperialist style” of nationalism (Ma Y., 2015), the heart of which was to protect the communist regime and territorial integrity. Since the late 1970s, Chinese government carried out “reform and open-up” policies and began to learn from the capitalistic economic system. However, the western pluralism and voting-based political democracy were still resisted by the nationalistic public in China (Ma L., 2015). The history of western invasions, combining with the governmental propaganda, result in that the nationalism in China is characterized by hostility to western countries and safeguarding China’s territorial integrity (Ma L., 2015). Thus, nationalism here are defined as the conviction of superiority of one’s country over other countries, especially including hostility to other countries.

Nationalism is usually a negative and even destructive ideology, which may lead to hostility, discrimination and national hubris. However, since nationalism could be a powerful political weapon to unite a nation (Liu and Zhou, 2019), it has been adopted by many countries, especially in times of crisis (Su and Shen, 2020; Zhang and Jamali, 2022). The nationalistic people in China are more patriotic, and more supportive of traditional Chinese social norms as well as existing social order, attaching importance to Confucianism and Marxism-Leninism (Ma L., 2015). Empirical research in China also found that stronger the nationalistic ideology was, more supportive of the Chinese government individuals would be (Ma and Zhang, 2017). Therefore, the H1c is suggested.

*H1c*: Nationalistic ideology positively predicts public support for official behaviors in COVID-19.

### 2.3 Behavioral factors: media diversity and echo chamber acts

Media use has been verified to influence people’s political attitude (Lu et al., 2016; Xu et al., 2022; Li and Liu, 2023; Qin et al., 2023). However, previous studies mainly classified media by its channel type, and explored the impacts of newspaper, television or Internet on political attitude (Willnat and Metzgar, 2012; Ji and Xiao, 2023). This was effective in a low-choice media environment. Whereas, in a high-choice media environment nowadays, the media usage becomes highly individualized and unique. People may choose to combine various and at times overlapping media, or use them individually. Thus, single media studies are difficult to represent the realistic context of a multiple media environment (Dubois and Blank, 2018). In consequence, the diversity and homogeneity of media contact behaviors, instead of use of different media types, are measured in this study.

#### 2.3.1 Media diversity

The high-choice media environment may generate two possible outcomes. Individuals may be exposed to various information and perspectives which are also diverse or they may select varied media in a way that produces echo chamber effects.

We use media diversity to represent the various information people may contact with in COVID-19, which is concerned with the number of media sources in a person’s media repertoire. However, few empirical studies have certified the influence of media diversity on support for government. Researchers only found that official and unofficial media had different effects on people’s viewpoints of government (Ma and Huang, 2023). For instance, it was found that the more Chinese were exposed to information from official media, the more likely they were to oppose democratic values and support the current government. On the contrary, the usage of unofficial media or foreign media led Chinese to identify with democratic values and oppose the government (Ma and Zhang, 2017; Yu and Wang, 2023). Because the uses of different media sources had significant impacts on Chinese attitude toward government, we hypothesize that media diversity might also significantly affect public support for official behaviors in COVID-19. However, due to the lack of empirical evidence, how media diversity affects pro-government attitude remains to be explored. Thus, H2a is stated as below:

*H2a*: Media diversity significantly predicts public support for official behaviors in COVID-19.

#### 2.3.2 Echo chamber acts

The idea of “echo chamber” is a metaphorical way to describe a situation where only certain ideas, information and beliefs are shared (Jamieson and Cappella, 2008; Sunstein, 2009; Li et al., 2022), in which setting people would only encounter things they already agree with.

We use echo chamber acts to represent the homogeneous information that people contact with in COVID-19. Though abundant media available online and offline is nowadays, individuals still prefer to consume opinion-reinforcing news over opinion-challenging ones (Garrett, 2009; Yuan and Wang, 2022). Furthermore, social media algorithms may also limit users’ exposure to diverse viewpoints. Once a user engages with opinion-reinforcing content, algorithmic filtering may constrain further exposure to a narrower, more closely aligned range of content (Stroud, 2010; Pariser, 2011). The emergence of an echo chamber may have serious negative consequences. Researchers worried that echo chambers might foster the adoption of more extreme opinions or ideological positions, and distort individual cognition of shared values of society (Hart et al., 2009; Cinelli et al., 2021; Pinto-Bustamante et al., 2023). However, empirical evidence about how echo chambers shape public attitude toward government is inconclusive. This paper hopes to verify the influences of echo chamber acts on public support for government in COVID-19 through empirical research.

Theoretically speaking, limited information strengthens preexisting biases, and encourages the adoption of more extreme viewpoints, which indicates the possible influences of echo chamber acts on public standpoints. In the meantime, the control over media by Chinese government makes it easier for Chinese people to access information that is supportive of government. If Chinese people tried not to contact with different information and avoided the impacts of echo chambers, they might be more likely to support official behaviors in COVID-19. Thus, H2b is suggested.

*H2b*: Echo chamber acts positively predict public support for official behaviors in COVID-19.

### 2.4 Environmental factors: social class

Except for personal and behavioral factors, environmental factors such as social class also shape people’s perceptions of government (Kraus et al., 2012).

Social class reflects an individual’s relative position on the ladder of social hierarchy, and guides patterns of thoughts, feelings and actions. For lower-class individuals, they are more dependent on external forces due to the lack of educational and economic resources, which leads to the formation of contextualist social cognitive tendency. In contrast, for upper-class individuals, with abundant material resources and elevated rank in society, they are free to pursue the interests they choose for themselves and tend to ignore the influences of situational factors, which causes solipsistic social cognitive tendency (Kraus et al., 2012). It was also found by empirical researches that individuals from lower-class were less likely to support the current social system (Yang et al., 2016), and the lower social class migrant workers were, the more likely they were to oppose the government (Liu et al., 2008). On the contrary, people with higher income and education level are more supportive of Chinese government’s actions (Ma and Huang, 2023). Therefore, the H3 is proposed:

*H3*: Social class positively predicts public support for official behaviors in COVID-19.

### 2.5 The mediating roles of echo chamber acts

According to triadic reciprocal determinism, the complex ways of reciprocal influences among personal, behavioral, and environmental factors encourage us to explore the possible mediating effects in our theoretical model.

Firstly, because individuals with high openness are fond of new information and trying new things, it is reasonable to conclude that individuals with openness personality are willing to avoid echo chambers. Also, when they are exposed to various information, they are more likely to change their opinions. On the contrary, conscientious individuals are relatively conservative and traditional, which means they are less likely to seek different information and avoid the influences of echo chambers. Thus, they are less likely to change their opinions about official behaviors. Therefore, echo chamber acts might have mediating effects on the relationships between openness\conscientiousness and public support for official behaviors in COVID-19.

*H4a*: Echo chamber acts mediate the effects of openness\conscientiousness on public support for official behaviors in COVID-19.

Secondly, as for the ideology, nationalism has been adopted as the main strategy for holding China together (Steele and Lynch, 2013). Because the media control is relatively strict in China, it can be speculated that Chinese domestic media are more inclined to express nationalistic ideology. As a result, the nationalistic public in China are more likely to consume opinion-reinforcing information and take echo chamber acts, which may further strengthen their supports for official behaviors. Thus, H4b is proposed:

*H4b*: Echo chamber acts mediate the effect of nationalistic ideology on public support for official behaviors in COVID-19.

Thirdly, existing empirical study verified the influence of media diversity on echo chamber acts. The more diverse media use was, the less echo chamber acts were conducted by respondents (Dubois and Blank, 2018). With the expansion of media diversity, individuals are more likely to be exposed to different viewpoints and avoid the effects of echo chambers, which may further encourage people to revise their original opinions. In other words, echo chamber acts might also have mediating effects on the relationship between media diversity and the dependent variable. Thus, the H4c is proposed:

*H4c*: Echo chamber acts mediate the effect of media diversity on public support for official behaviors in COVID-19.

At last, compared with elites from higher social class, lower-social class individuals are more likely to immerse themselves in the homogenized information environment and take echo chamber actions (Yu and Fang, 2020), which might further affect one’s attitude toward government. Thus, we speculate the H4d:

*H4d*: Echo chamber acts mediate the effect of social class on public support for official behaviors in COVID-19.

**Figure 1:**
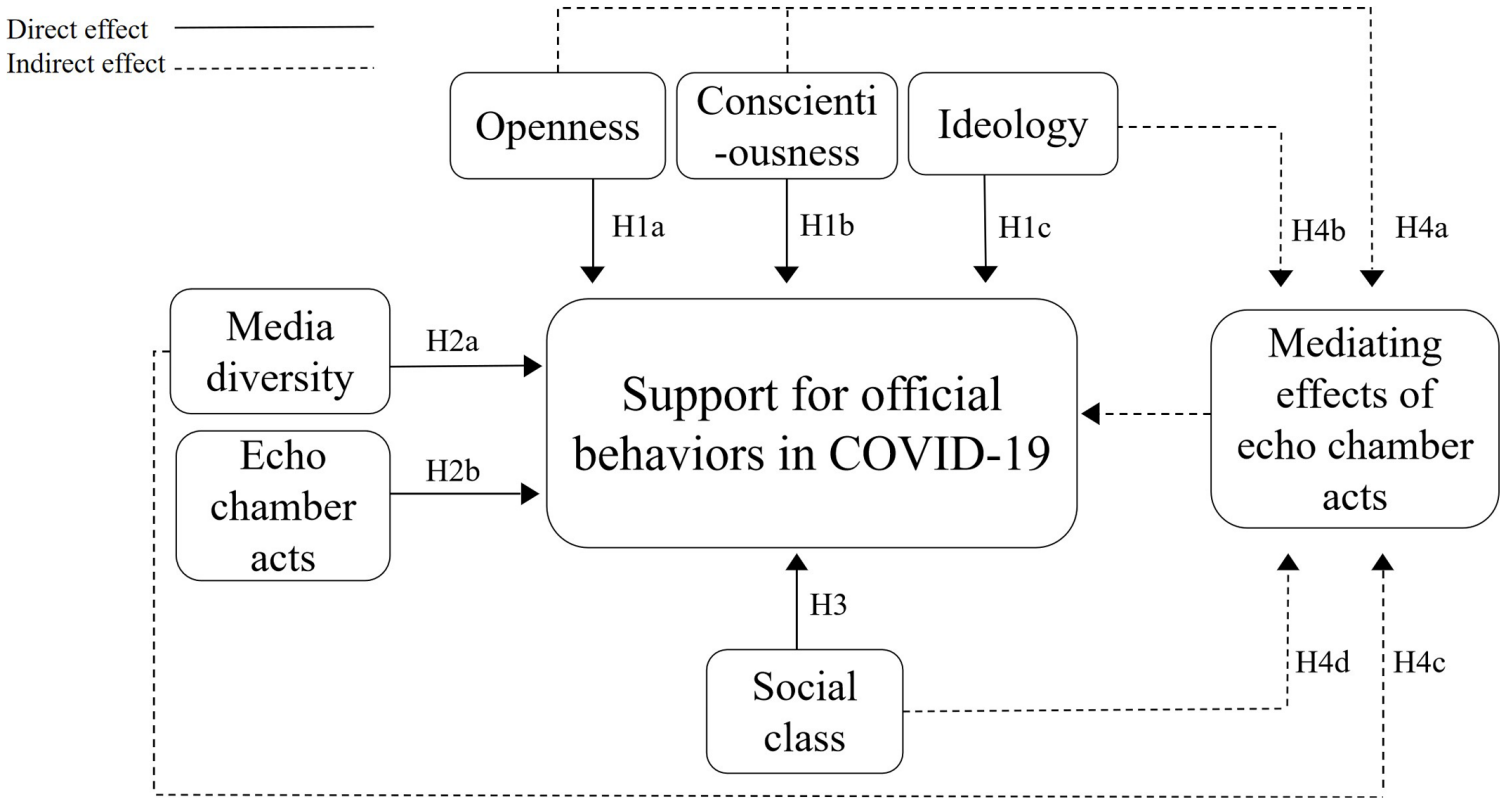
The theoretical model.

Based on the triadic reciprocal determinism and the discussions above, we propose a theoretical model as shown in Figure 1.

## 3 Research methods

To explore the proposed model, this study used a self-report questionnaire survey to measure the personality, ideology, media diversity, echo chamber acts, social class, and public support for official behaviors in COVID-19.

The study was reviewed and approved by the Academic Ethics Committee, School of Journalism and Communication, Beijing Normal University (Approval No. BNUJ&C20231116001). All participants provided signed consent for participation.

### 3.1 Sample

The questionnaire data was collected online through the database of “Shanghai Zhongyan Technology Co., LTD” between November 13th and 17th 2020. The company has established a database of about 40 million quantitative survey respondents in China. Participants of this survey were limited to Wuhan adult citizens aged 18–65. Firstly, the questionnaires were issued to citizens with IP addresses in Wuhan through the company’s database. Secondly, participants were asked whether or not they were 18–65 years old and lived in Wuhan from December 2019 when the COVID-19 out-broke in Wuhan. Only participants who met these two criteria could continue to fill out the questionnaire.

Since Wuhan was the first city to bear the brunt of the COVID-19 pandemic in China, people in Wuhan were close to COVID-19 and paid more attention to the coping measures conducted by the government compared with residents in other cities. The incident relevant to Doctor Li Wenliang in the dependent variable also happened in Wuhan. Therefore, Wuhan citizens had a better understanding of the items that we measured for our dependent variable, which could ensure the validity of the questionnaire survey.

It was found that gender, age, and educational background were the main demographic variables that impacted political attitude (Ma and Zhang, 2017). In order to avoid the deviation of research conclusions caused by sample bias, the quota sampling method was adopted according to the gender and age structure of Wuhan population, as well as the education structure of the urban population in China. To reduce sample bias, questionnaires were randomly distributed in the survey database of Shanghai Zhongyan Technology Company according to the quota ratio of gender, age, and educational background.

Referring to the *2018 Wuhan Statistical Yearbook* published by the Wuhan Bureau of Statistics, in the adult population aged 18–65 in Wuhan City, males accounted for 50.48% and females accounted for 49.52%, each accounting for about 50%. Also, people aged 18–25 accounted for 11.70%, people aged 26–35 accounted for 26.43%, people aged 36–45 accounted for 19.78%, people aged 46–55 accounted for 23.73%, and people aged 56–65 accounted for 18.36%. Moreover, based on the data from *1% National Sample Survey* published by National Bureau of Statistics of China in 2016, in the urban population aged 18–65 in China, the proportion of below primary school education was 2.31%, the proportion of primary school education was 11.36%, the proportion of junior high school education was 32.39%, the proportion of high school education was 24.04%, the proportion of junior college education was 14.03%, the proportion of bachelor’s degree was 14.12%, and the proportion of master’s degree or above was 1.75%.

A total of 800 valid questionnaires were collected in our study. The invalid data are stored on the computer server in School of Journalism and Communication, Beijing Normal University. As shown in Table 1, the population structure of our sample is close to that of Wuhan City.

**Table 1:** Demographic information (*N* = 800).

	Category	Numbers in our sample	Percentage in our sample	Percentage in statistics Bureau data
Gender	Male	400	50%	50.48%
	Female	400	50%	49.52%
Age	18–25	104	13%	11.70%
	26–35	216	27%	26.43%
	36–45	160	20%	19.78%
	46–55	192	24%	23.73%
	56–65	128	16%	18.36%
Education	Below primary school	16	2%	2.31%
	Primary school	88	11%	11.36%
	Junior high school	256	32%	32.39%
	High school	192	24%	24.04%
	Junior college	112	14%	14.03%
	Bachelor’s degree	112	14%	14.12%
	Master’s degree or above	24	3%	1.75%

### 3.2 Measures

#### 3.2.1 Personality

The openness and conscientiousness were assessed with the use of “Chinese version of the Big Five personality scale” (Li, 2013) (assessments were ranged between 1 = “Strongly disagree” to 5 = “Strongly agree”; Openness *min* = 1, *max* = 5, *M* = 3.71, *SD* = 0.661, Cronbach’s α = 0.797; Conscientiousness *min* = 2, *max* = 5, *M* = 4.03, *SD* = 0.409, Cronbach’s *α* = 0.506). The scale has been verified to have good reliability and validity in the context of China (Li, 2013; Xia et al., 2013). The measurement items included “I think I am open to new things and always have new ideas”; “I think I am trustworthy and self-disciplined.” In the analysis, the averages of measurement items were calculated to represent openness and conscientiousness.

#### 3.2.2 Ideology

The ideological scale developed by Ma and Zhang (2017) in the context of Chinese culture was adopted to measure the nationalistic ideology (assessments were ranged between 1 = “Strongly disagree” to 5 = “Strongly agree”; *min* = 1, *max* = 5, *M* = 3.74, *SD* = 0.728, Cronbach’s α = 0.716). The scale also has been proved to be reliable and valid in China (Ma and Wang, 2015). The measurement items included “China’s diplomatic issues, such as territorial and trade disputes, are provoked by other countries in the first place” etc. Also, the average of measurement items represents ideology. Based on the design of this tool, the higher the average score, the more nationalistic ideology espoused.

#### 3.2.3 Media diversity

To measure media diversity, the questionnaire asked “When looking for information about COVID-19, how often do you contact with news from party media in China/ commercial media in China/ self-media in China/ foreign media organization/ self-media outside China.” For each type of media source, the questionnaire listed typical media names to ensure the validity of respondents’ answers. Responses were measured on a 5-category Likert scale from 0 = “Never” to 4 = “Very often” (*min* = 0.50, *max* = 3.33, *M* = 1.96, *SD* = 0.263). The average score of all items represents media diversity. It is understandable that the internal consistency of media diversity is low because it measures respondents’ exposure to highly differentiated media sources. Thus, the Cronbach’s α value is not reported for media diversity.

#### 3.2.4 Echo chamber acts

The scale of Dubois and Blank (2018) was used to measure echo chamber acts (assessments were ranged between 0 = “Never” to 4 = “Very often”; *min* = 0.33, *max* = 4, *M* = 2.09, *SD* = 0.448, Cronbach’s *α* = 0.661). Items included “How often do you read something you disagree with in COVID-19.” In the analysis, the values of items were reversed and the average score of reversed items was adopted to represent echo chamber acts. Also, reliability and validity of the scale have been confirmed by previous research in China (Ding et al., 2020).

#### 3.2.5 Social class

Social class was typically assessed by objective material resources using reports of family income, education, and occupation status. This paper adopted Li Qiang’s “Social class scale of residents in China’s big cities” (Li, 2019) to measure social class (*min* = 1, *max* = 7.33, *M* = 4.40, *SD* = 1.27, Cronbach’s α = 0.673). The average of each measurement item represents the social class. The scale has been verified to be reliable and valid in the context of China’s big city (Li, 2019; Tan et al., 2020).

#### 3.2.6 Public support for official behaviors in COVID-19

To assess the dependent variable in this study, the author sorted out six official behaviors that have been widely discussed in COVID-19. A 5-category Likert scale 1 = “Strongly disagree” to 5 = “Strongly agree” was used to measure the dependent variable (*min* = 2, *max* = 5, *M* = 4.12, *SD* = 0.230, Cronbach’s α = 0.621). The items of dependent variable are shown in Table 2. The average of each item represents the dependent variable. And higher the average score, the more supportive of official behaviors in COVID-19 the respondents will be.

**Table 2:** Items of public support for official behaviors in COVID-19.

Items	Min	Max	Mean	SD
Building roadblocks to seal off villages and towns is an epidemic prevention and control measure that China must take;	3	5	4.31	0.439
The Chinese government has brought the epidemic under control and achieved positive GDP growth, which reflected China’s superiority;	1	5	4.34	0.527
The Chinese government responded positively to the supervision by public opinion in COVID-19;	2	5	4.11	0.515
The “Investigation of Doctor Li Wenliang Incident” issued by the investigation team from the national supervisory commission has well responded to the concerns of the public;	1	5	4.09	0.683
Other countries should learn from the Chinese government in epidemic prevention and control;	1	5	3.86	0.932
Unlike the Chinese government, the “herd immunity” policy suggested by the British government was irresponsible.	1	5	4.03	0.901

## 4 Results

### 4.1 Data analysis method

Structural equation model (SEM) is the most efficient technique for estimating a series of separate multiple regressions (Sun and Zhang, 2021). Therefore, SEM was used to test the theoretical model. All calculations were completed by AMOS 23.0 software.

The medium sample size (200<sample size<1,000) and the multivariate normality in the data of this study are more suitable for the maximum likelihood (ML) method (Tabachnick and Fidell, 2007). Hence, The ML method was used for calculations.

The results of assessment of normality in AMOS (Table 3) reveal that the data set is close to normal because the skew numbers of all variables are less than 3, and the kurtosis values of all variables are less than 8 (Kline, 1998).

**Table 3:** Assessment of normality.

Variable	min	max	skew	critical ratio	kurtosis	critical ratio
Openness	1.000	5.000	−0.865	−9.986	0.718	4.144
Conscientiousness	2.000	5.000	−0.657	−7.586	0.550	3.178
Ideology	1.000	5.000	−1.039	−11.998	0.804	4.641
Media diversity	0.500	3.333	−0.183	−2.118	−0.121	−0.699
Echo chamber acts	0.333	4.000	−0.111	−1.276	−0.205	−1.181
Social class	1.000	7.333	0.105	1.209	−0.341	−1.971
Public support for official behaviors in COVID-19	2.000	5.000	−0.822	−9.495	0.730	4.214
Multivariate					5.025	6.331

### 4.2 Measurement model analysis

We examined two types of validity such as convergent validity and discriminant validity to evaluate the measurement model. Convergent validity is confirmed by examining standardized factor loading and average variance extracted (AVE). As shown in Table 4, most of the standardized factor loading values are above the recommended value of 0.5 (Bagozzi and Yi, 1988). The AVE values of openness, ideology, and social class are higher than 0.5, which indicates fairly good convergent validity (Chin, 1998). However, the AVE values of conscientiousness, echo chamber acts and public support for official behaviors in COVID-19 are lower than 0.5.

**Table 4:** Convergent validity.

Constructs and items	Factor loading	*R*^2^	Error	CR	AVE
Openness				0.801	0.668
I think I am open to new things and always having new ideas	0.857	0.734	0.266		
I’d rather like to follow the routine and dislike innovation	0.776	0.602	0.398		
Conscientiousness				0.551	0.394
I think I am trustworthy and self-disciplined	0.460	0.212	0.788		
I think I am ill-organized and careless	0.759	0.576	0.424		
Ideology				0.743	0.502
China’s diplomatic issues, such as territorial and trade disputes, are provoked by other countries in the first place	0.651	0.423	0.577		
If conditions permit, Taiwan should be reunified by force	0.537	0.288	0.712		
Foreign hostile forces are the causes of many China’s problems	0.891	0.794	0.206		
Echo chamber acts				0.664	0.397
Read something you disagreed with	0.610	0.373	0.627		
Checked a news source that was different from what you normally read	0.666	0.444	0.556		
Discovered something that changed your opinion	0.613	0.376	0.624		
Social class				0.807	0.584
Education	0.682	0.465	0.535		
Monthly income per family member	0.848	0.718	0.282		
Occupational status	0.754	0.569	0.431		
Public support for official behaviors in COVID-19				0.642	0.230
Building roadblocks is indispensable in China	0.517	0.268	0.713		
The positive GDP growth reflected China’s superiority	0.479	0.229	0.777		
Chinese government responded positively to supervision	0.458	0.210	0.776		
Investigation of Li incident has well responded to public concerns	0.458	0.210	0.798		
Others should learn from the Chinese government	0.455	0.207	0.801		
The “herd immunity” policy was irresponsible	0.508	0.258	0.739		

The reverse item may be the main reason of the low AVE value of conscientiousness. And the relatively low AVE value of echo chamber acts may be because that the scale was developed by Canadian and British scholars and might not be applicable to the Chinese context. In addition, the measurement items of support for official behaviors in COVID-19 were selected by the author according to the heated incidents in COVID-19 in China. Though related to the government every incident is, the nature of various incidents is slightly different from each other, which may decrease the AVE value. Therefore, future research should conduct a pre-survey to modify scales before conducting a formal survey.

Discriminant validity is the extent to which each latent variable is distinct from all other variables in the model (Chin, 2010). Table 5 informs that square roots of AVE values (shown on the diagonal of the correlation matrix) are larger than the inter-construct correlations (presented on the off-diagonal elements), suggesting that each measurement item is better explained by its intended construct than by other constructs (Kline, 1998).

**Table 5:** Discriminant validity.

	OPE	CON	IDE	MD	ECA	SC	PSOB-C19
OPE	**0.817**						
CON	0.266^**^	**0.628**					
IDE	0.051	0.203^*^	**0.709**				
MD	0.158^**^	0.049	−0.042	**0.387**			
ECA	−0.141^**^	0.065	0.115^**^	−0.259^**^	**0.630**		
SC	0.154^**^	0.059	−0.012	0.176^**^	−0.128^**^	**0.764**	
PSOB-C19	0.102^**^	0.294^*^	0.446^**^	0.026	0.160^**^	0.058	**0.480**

OPE, openness; CON, conscientiousness; IDE, ideology; MD, media diversity; ECA, echo chamber acts; SC, social class; PSOB-C19, public support for official behaviors in COVID-19. ^**^and ^*^indicate significant correlation at the 0.05 and 0.1 level. Bold values indicate the square roots of AVE values.

### 4.3 Structural model analysis

**Figure 2:**
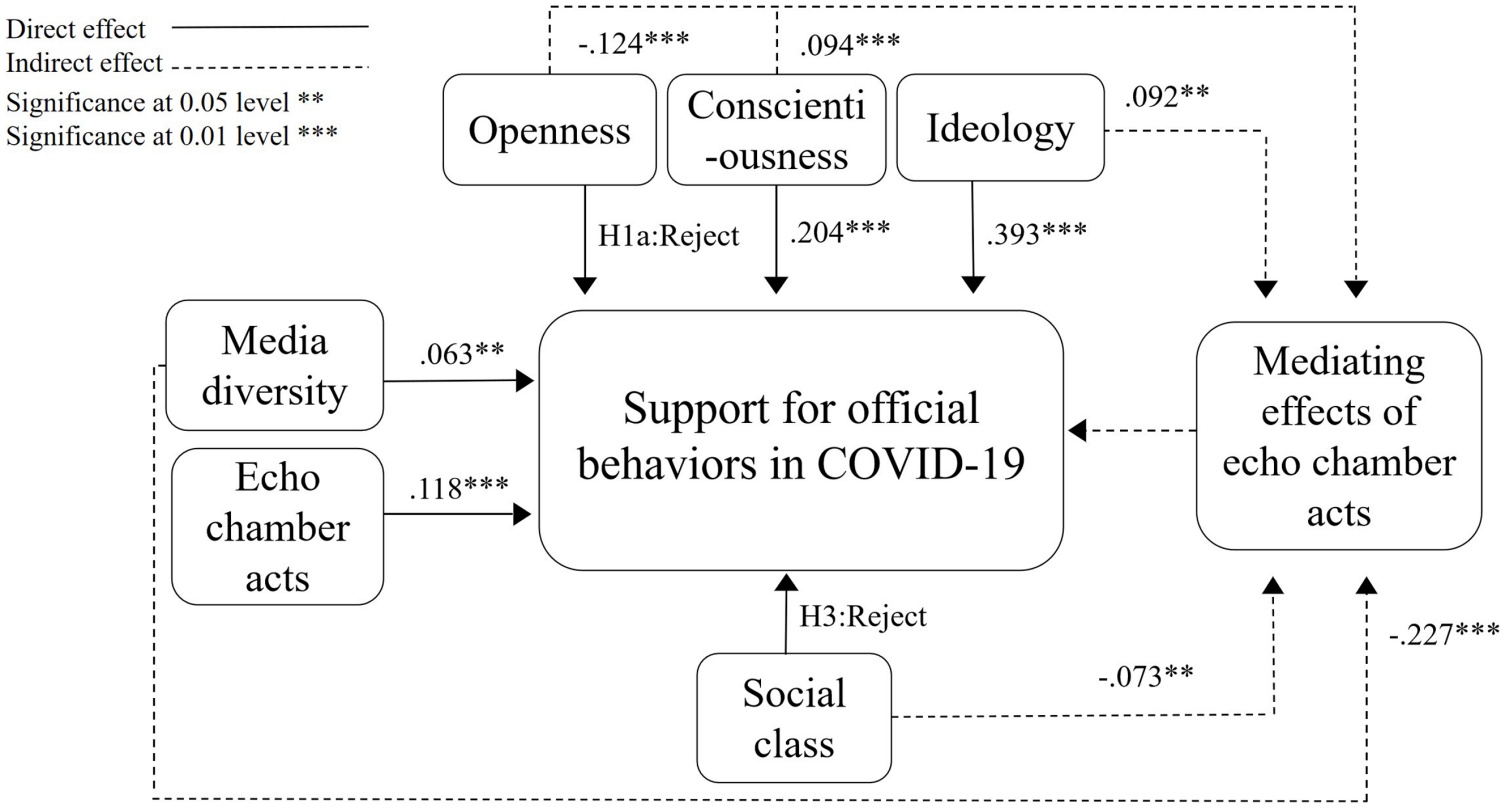
Standardized estimates of influencing paths.

As shown in Figure 2, a structural model was built with public support for official behaviors in COVID-19 as the dependent variable. The chi-square value of the overall model is 4.374. And the significance probability value *p* is 0.112, which do not reach the significant level of 0.05, indicating that the model assumed by path analysis can be supported. Also, CFI = 0.995 > 0.9, RMSEA = 0.039 < 0.05, indicating a good fit of the model.

#### 4.3.1 Direct determinants of public support for official behaviors in COVID-19

For personal factors, conscientiousness [Standardized *β* = 0.204, *p* = 0.002<0.01, 95% CI (0.132, 0.274), S.E. = 0.036] and ideology [Standardized *β* = 0.393, *p* = 0.002<0.01, 95% CI (0.323, 0.459), S.E. = 0.035] are found to exert significantly positive impacts on support for governmental actions in COVID-19. Thus, the H1b and H1c are supported, and the H1a is rejected.

For behavioral factors, media diversity [Standardized *β* = 0.063, *p* = 0.031<0.05, 95% CI (0.005, 0.125), S.E. = 0.029] and echo chamber acts [Standardized *β* = 0.118, *p* = 0.002<0.01, 95% CI (0.056, 0.175), S.E. = 0.030] significantly and positively influence support for governmental responses to COVID-19, supporting the H2a and H2b.

For environmental factors, the relationship between social class and public support for official behaviors is statistically insignificant. Thus, H3 is rejected.

The results suggest that individuals with higher level of conscientiousness, nationalistic ideology, media diversity, and echo chamber acts are more likely to identify with the official behaviors in COVID-19. The percentage of variance of dependent variable that can be explained by the model is 25.6%.

#### 4.3.2 Direct determinants of echo chamber acts

For personal factors, openness [Standardized *β* = −0.124, *p* = 0.003<0.01, 95% CI (−0.185, −0.052), S.E. = 0.034] exerts significantly negative effect on echo chamber acts. Conscientiousness [Standardized *β* = 0.094, *p* = 0.007<0.01, 95% CI (0.029, 0.161), S.E. = 0.034] and nationalistic ideology [Standardized *β* = 0.092, *p* = 0.019<0.05, 95% CI (0.012, 0.156), S.E. = 0.037] have positive effects on echo chamber acts.

For behavioral factors, media diversity [Standardized *β* = −0.227, *p* = 0.002<0.01, 95% CI (−0.292, −0.165), S.E. = 0.032] is found to exert significantly negative impact on echo chamber acts.

For environmental factors, social class [Standardized *β* = −0.073, *p* = 0.035<0.05, 95% CI (−0.144, −0.005), S.E. = 0.034] is verified to significantly and negatively influence echo chamber acts.

The results suggest that individuals with higher level of conscientiousness and nationalistic ideology are more likely to perform echo chamber acts. However, individuals with higher level of openness, media diversity, and social class are more likely to resist echo chamber acts in COVID-19. The percentage of variance of echo chamber acts that can be explained by openness, conscientiousness, ideology, media diversity and social class is 10.1%.

#### 4.3.3 Mediating roles of echo chamber acts

This study used bootstrapping procedures in AMOS to test the mediating effects (Table 6). The echo chamber acts play important mediating roles between the relationships of openness/conscientiousness/ideology/media diversity/social class and public support for official behaviors in COVID-19.

**Table 6:** The mediating effects of echo chamber acts.

	Standardized direct effects	Standardized indirect effects	Standardized total effects
OPE → ECA → PSOB-C19	0.000	−0.015^**^ (−0.028, −0.006)	−0.015^**^ (−0.028, −0.006)
CON→ECA → PSOB-C19	0.204^**^ (0.132, 0.274)	0.011^**^ (0.003, 0.025)	0.215^**^ (0.141, 0.283)
IDE → ECA → PSOB-C19	0.393^**^ (0.323, 0.459)	0.011^**^ (0.003, 0.022)	0.404^**^ (0.331, 0.469)
MD → ECA → PSOB-C19	0.063^**^ (0.005, 0.125)	−0.027^**^ (−0.044, −0.013)	0.036 (−0.021, 0.094)
SC → ECA → PSOB-C19	0.000	−0.009^**^ (−0.020, −0.001)	−0.009^**^ (−0.020, −0.001)

OPE, openness; CON, conscientiousness; IDE, ideology; MD, media diversity; ECA, echo chamber acts; SC, social class; PSOB-C19, public support for official behaviors in COVID-19. The values in round brackets are 95% confidence intervals; ^**^indicates significance at 0.05 level; Bootstrap = 1,000.

The indirect effects of echo chamber acts are all significant. Thus, the H4a, H4b, H4c and H4d are all supported. Furthermore, the results suggest that individuals with lower degree of openness, higher level of conscientiousness, stronger tendency toward nationalistic ideology, lower diversity of media exposure, and lower social class are more likely to perform echo chamber acts, and thus are more likely to support the official behaviors in COVID-19.

## 5 Conclusion and discussion

With the emergence of opinion polarization phenomenon in China’s public opinion field, it is vital to analyzing the underlying causal mechanisms of political attitude in China. Combining with triadic reciprocal determinism, we aimed to evaluate the determinants of public support for governmental behaviors in COVID-19, and a comprehensive theoretical model with personal, behavioral, and environmental factors was constructed. Based on the results, this study presents the following four key findings:

Firstly, congruent with some previous studies, the results of this study support the hypothesis that conscientiousness positively predicts identification with authority and government (Carney et al., 2008; Gerber et al., 2010). In an event full of uncertainties and threats like the COVID-19 pandemic, conscientious individuals are more eager for order to reduce uncertainties, so they tend to support the official authority and the coercive measures taken by the Chinese government. Study in America also found that conscientiousness was positively associated with following government guidelines at the start of the COVID-19 pandemic (Bogg and Milad, 2020). However, the influence of conscientiousness on pro-government attitude is still inconclusive. For instance, it was found that conscientiousness had small negative association with vaccine intention and vaccination (Bogg et al., 2023; Godfrey et al., 2024), or had little impact on mask-wearing behaviors proposed by American government (Milad and Bogg, 2021).
Secondly, in alignment with existing studies (Ma and Lu, 2022; Ma and Lu, 2023), the empirical results of this study also indicate that nationalistic ideology positively affect people’s support for official behaviors. To cope with the death anxiety caused by the COVID-19 pandemic, people were predisposed to ideological defense, resulting in cohesion with individuals who validated their beliefs and hostility toward those who threatened them (Ma and Lu, 2019). For nationalistic individuals, supporting the Chinese government is aligned with their ideology. Thus, nationalistic individuals tend to support official behaviors in COVID-19.
Thirdly, the results of this study also confirm the positive effects of echo chamber acts on public support for government in COVID-19, which has not been verified by empirical survey. Reflecting the subtle and delicate control of media, Chinese news content in both news media and social media has pro-government features (Zhang, 2022; Zhang and Xu, 2023) in which expressions of nationalism are the norm (Zhang, 2020). In consequence, it is easier for Chinese citizens to get access to information that supports the government. If they were not exposed to information from other sources and tried to reduce echo chamber acts, their supports for the official behaviors would continue to grow.
Fourthly, it is found that echo chamber acts play important mediating roles in our theoretical model. Personal factors like personality and ideology, behavioral factors such as media diversity, and environmental factors like social class are all verified to have significant impacts on echo chamber acts. And echo chamber acts would further influence public support for governmental actions in COVID-19. High levels of open personality, media diversity, and social class are conducive to avoiding echo chambers, which are consistent with the conclusions of previous researches (Li et al., 2013; Dubois and Blank, 2018; Yu and Fang, 2020). On the contrary, with higher level of conscientious personality and nationalistic ideology, individuals are more susceptible to echo chambers, which would strengthen their support for official behaviors. Interestingly, the results highlight that individuals with nationalistic ideology would take more echo chamber acts, and then would be more likely to stress the superiority of coping measures adopted by the Chinese government and despise measures taken by other countries in COVID-19. This would further strengthen their nationalistic tendency, leading to the polarization of one’s viewpoint. Under the influences of echo chambers, people who initially hold neutral opinions may also develop extreme standpoints gradually.

## 6 Practical implication

It is confirmed in this study that individuals with lower level of conscientiousness, nationalistic ideology, and media diversity are more likely to doubt the measures taken by the government in pandemic. Though worried about pandemic these people are, they are more anxious about the possible negative consequences of governmental actions. From the perspective of practice, government should take several measures to engender trust in public health crisis like COVID-19. Firstly, the benefits of emergency measures should be emphasized to reduce the anxiety in public. Secondly, since people with low-level conscientiousness and nationalistic ideology are more worried about official behaviors, it is necessary to continually reevaluate conditions and rescind quarantine measures at appropriate time. The restrictions on behaviors of enforcement officers should also be explicit to reduce anxiety of this part of the population. Thirdly, individuals with high-level conscientiousness have the features of “order, structure, closure, certainty, consistency, simplicity, and familiarity” (Jost et al., 2014). Therefore, for this part of the public, governmental messaging should be directly linked to an end to perceived disruptions and a return to simplicity, especially the return of one’s familiar lifestyle (Bogg et al., 2023). Finally, media diversity impact public support for government in COVID-19. Thus, it is important to use as many media platforms as possible to convey policies and promote public understanding of emergency measures in public health crisis. Variation in confidence in governments and institutions is a common theme across borders. Future studies could focus on the topic and propose more approaches to enhance public trust.

The significance of echo chamber acts is emphasized in this study. Although echo chamber acts can enhance public support for official behaviors in health crisis events, which would be beneficial to implement control measures and bring the epidemic under control, we should also be aware of the negative effects of echo chambers. The “strongly support” of the government’s position results from echo chambers is “false consensus” that may lead to extreme attitude (Ma and Huang, 2023) and threaten democracies by limiting political discussions (Prior, 2007; Ksiazek et al., 2010). To construct a healthy and normal public opinion field in health risk crisis, it is vital to reducing echo chamber acts and encouraging people with different views to talk with each other.

Since the Internet platforms have become the main channels for people to access information, we propose to focus on using Internet platform algorithms to reduce echo chamber acts (Wiard et al., 2022). For Internet platforms like Tik Tok and Toutiao.com, an echo chamber user identification mechanism should be built based on users’ personality, ideology, media contact behaviors and social class. A person with conscientious personality, nationalistic ideology, low-level media diversity, and low social class can be identified and marked as “person susceptible to echo chambers,” while a person with open personality, high-level media diversity, and high social class can be labeled as “person resistant to echo chambers.” For individuals susceptible to echo chambers, Internet platforms can increase the distribution weight of heterogeneous content in personalized recommendation algorithm. In addition, the study results confirm that media source diversity can effectively mitigate echo chamber acts. In consequence, the recommendation system should capture and tag the various media sources as completely as possible. And the recommendation algorithm should consider the diversity of media sources when distributes heterogeneous content.

While contributing insight to determinants of public cognition in China, the research results also have significance for the construction of healthy and normal public opinion field in China. This provides a good starting point for further research. But the findings of study have to be seen in light of some limitations.

Firstly, this study was conducted in the context of China, in which some special circumstances might affect the extensibility of the research conclusions. For example, the media control in China is relatively strict, and most of the media messages that can be accessed are in defense of the government. This may be the main reason why echo chamber acts positively affect public support for governmental behaviors in China. However, the media environments in other countries are different from China. Thus, future studies are suggested to investigate the factors influencing public support for governmental actions in different countries.

Secondly, the AVE values of echo chamber acts and dependent variable are relatively low in this study. This is because the measurement items were developed by scholars in other countries rather than in China, or they were selected by the author. Therefore, for future studies, it is necessary to develop echo chamber acts scale that is suitable for the country being studied. Also, for self-made scale, a pre-survey must be conducted to modify and verify the scale.

Thirdly, the reliability of the conscientiousness scale is relatively low, which may be resulted from the reversed item in the scale. Also, the openness and conscientiousness are measured by two-item scale, and the ideology as well as echo chamber acts are measured by three-item scale, which may also cause deviation in measurement.

## Data availability statement

The original contributions presented in the study are included in the article/supplementary material, further inquiries can be directed to the corresponding author.

## Ethics statement

The studies involving humans were approved by Academic Ethics Committee, School of Journalism and Communication, Beijing Normal University (Approval No. BNUJ&C20231116001). The studies were conducted in accordance with the local legislation and institutional requirements. The participants provided their written informed consent to participate in this study.

## Author contributions

BM: Validation, Resources, Project administration, Data curation, Writing – review & editing, Writing – original draft, Visualization, Software, Methodology, Investigation, Funding acquisition, Formal analysis, Conceptualization. HD: Project administration, Writing – review & editing, Supervision, Methodology, Investigation, Funding acquisition, Conceptualization.

## References

[ref1] Albert B. (1986). The explanatory and predictive scope of self-efficacy theory. J. Soc. Clin. Psychol. 4, 359–373. doi: 10.1521/jscp.1986.4.3.359

[ref2] Albert B. (2001). Social cognitive theory: an agentic perspective. Annu. Rev. Psychol. 52, 1–26. doi: 10.1146/annurev.psych.52.1.1 11148297

[ref3] Albert B. Forest J. (1991). Self-regulatory mechanisms governing the impact of social comparison on complex decision making. J. Pers. Soc. Psychol. 60, 941–951. doi: 10.1037/0022-3514.60.6.941

[ref4] Bagozzi R. P. Yi Y. (1988). On the evaluation of structural equation models. J. Acad. Mark. Sci. 16, 74–94. doi: 10.1007/BF02723327

[ref5] Bavel J. V. Pereira A. (2018). The partisan brain: an identity-based model of political belief. PsyArXiv 22, 213–224. doi: 10.1016/j.tics.2018.01.004 29475636

[ref6] Beattie P. Chen R. Bettache K. (2022). When left is right and right is left: the psychological correlates of political ideology in China. Polit. Psychol. 43, 457–488. doi: 10.1111/pops.12776

[ref7] Bogg T. Milad E. (2020). Demographic, personality, and social cognition correlates of coronavirus guideline adherence in a US sample. Health Psychol. 39, 1026–1036. doi: 10.1037/hea0000891, PMID: 33252928

[ref8] Bogg T. Milad E. Godfrey O. (2023). COVID-19 vaccine intention: prospective and concurrent tests of a disposition-belief-motivation framework. Health Psychol. 42, 521–530. doi: 10.1037/hea0001200, PMID: 35759005

[ref9] Carney D. R. Jost J. T. Gosling S. D. Potter J. (2008). The secret lives of liberals and conservatives: personality profiles, interaction styles, and the things they leave behind. Polit. Psychol. 29, 807–840. doi: 10.1111/j.1467-9221.2008.00668.x

[ref10] Chin W. W. (1998). The partial least squares approach to structural equation modeling. Mod. Methods Bus. Res. 295, 295–336,

[ref11] Chin W. W. (2010). “How to write up and report PLS analyses” in Handbook of Partial Least Squares. eds. Esposito Vinzi V. Chin W. W. Henseler J. Wang H. (London: Springer), 655–690.

[ref12] Cinelli M. De Francisci Morales G. Galeazzi A. Quattrociocchi W. Starnini M. (2021). The Echo chamber effect on social media. Proc. Natl. Acad. Sci. 118:e2023301118. doi: 10.1073/pnas.2023301118, PMID: 33622786 PMC7936330

[ref13] Ding H. Miao B. Wang J. (2020). Behavior and environment: a study on the influencing factors of issues cognition in China. Contemp. Commun. 5, 28–33,

[ref14] Dubois E. Blank G. (2018). The echo chamber is overstated: the moderating effect of political interest and diverse media. Inf. Commun. Soc. 21, 729–745. doi: 10.1080/1369118X.2018.1428656

[ref15] Garrett R. K. (2009). Echo chambers online? Politically motivated selective exposure among internet news users. J. Comput. Mediat. Commun. 14, 265–285. doi: 10.1111/j.1083-6101.2009.01440.x

[ref16] Gerber A. S. Huber G. A. Doherty D. Dowling C. M. (2011). The big five personality traits in the political arena. Annu. Rev. Polit. Sci. 14, 265–287. doi: 10.1146/annurev-polisci-051010-111659

[ref17] Gerber A. S. Huber G. A. Doherty D. Dowling C. M. Ha S. E. (2010). Personality and political attitudes: relationships across issue domains and political contexts. Am. Polit. Sci. Rev. 104, 111–133. doi: 10.1017/S0003055410000031

[ref18] Godfrey O. Bogg T. Milad E. (2024). A psychosocial model of COVID-19 vaccination: antecedent and concurrent effects of demographics, traits, political beliefs, vaccine intention, information sources, mandates, and flu vaccine history. Ann. Behav. Med. 58, 12–21. doi: 10.1093/abm/kaad043, PMID: 37540839

[ref19] Hart W. Albarracin D. Eagly A. H. Brechan I. Lindberg M. J. Merrill L. (2009). Feeling validated versus being correct: a meta-analysis of selective exposure to information. Psychol. Bull. 135, 555–588. doi: 10.1037/a0015701, PMID: 19586162 PMC4797953

[ref20] Hiel A. V. Pandelaere M. Duriez B. (2004). The impact of need for closure on conservative beliefs and racism: differential mediation by authoritarian submission and authoritarian dominance. Person. Soc. Psychol. Bull. 30, 824–837. doi: 10.1177/0146167204264333, PMID: 15307224

[ref21] Hu R. Lin Z. (2022). Research on subjective class cognition, social capital and social governance efficacy of urban and rural residents. J. Univ. Chin. Acad. Soc. Sci. 42, 109–147,

[ref22] Jamieson K. Cappella J. (2008). Echo chamber: Rush Limbaugh and the conservative media establishment. London: Oxford UP.

[ref23] Ji N. Xiao W. (2023). How does media access affect political trust? -- empirical research based on the world values survey. J. Guangxi Normal Univ. 59, 83–97. doi: 10.16088/j.issn.1001-6597.2023.01.008

[ref24] Jost J. Federico C. Napier J. (2009). Political ideology: its structure, functions, and elective affinities. Annu. Rev. Psychol. 60, 307–337. doi: 10.1146/annurev.psych.60.110707.163600, PMID: 19035826

[ref25] Jost J. Glaser J. Kruglanski A. Sulloway F. (2003). Political conservatism as motivated social cognition. Psychol. Bull. 129, 339–375. doi: 10.1037/0033-2909.129.3.339 12784934

[ref26] Jost J. T. Noorbaloochi S. Van Bavel J. J. (2014). The “chicken-and-egg” problem in political neuroscience. Behav. Brain Sci. 37, 317–318. doi: 10.1017/S0140525X13002616, PMID: 24970439

[ref27] Kahan D. M. (2017). Misconceptions, misinformation, and the logic of identity-protective cognition. SSRN Electron. J. doi: 10.2139/ssrn.2973067

[ref28] Kline R. B. (1998). Principles and practice of structural equation modeling. New York: Guilford Press.

[ref29] Kosterman R. Feshbach S. (1989). Toward a measure of patriotic and nationalistic attitudes. Polit. Psychol. 10, 257–274. doi: 10.2307/3791647

[ref30] Kraus M. W. Piff P. K. Mendoza-Denton R. Rheinschmidt M. L. Keltner D. (2012). Social class, solipsism, and contextualism: how the rich are different from the poor. Psychol. Rev. 119, 546–572. doi: 10.1037/a0028756, PMID: 22775498

[ref31] Kruglanski A. Boyatzi L. (2012). The psychology of closed and open mindedness, rationality, and democracy. Crit. Rev. 24, 217–232. doi: 10.1080/08913811.2012.711023

[ref32] Ksiazek T. B. Malthouse E. C. Webster J. G. (2010). News-seekers and avoiders: exploring patterns of Total news consumption across media and the relationship to civic participation. J. Broadcast. Electron. Media 54, 551–568. doi: 10.1080/08838151.2010.519808

[ref33] Li J. (2013). Reliability and validity test of Chinese 10-item big five personality inventory (TIPI-C). Chin. J. Health Psychol. 21, 1688–1692. doi: 10.13342/j.cnki.cjhp.2013.11.008

[ref34] Li Q. (2019). Social stratification in contemporary China. Beijing: Life Bookstore Publishing Co., LTD.

[ref35] Li N. Gao C. Piao J. Huang X. Yue A. Zhou L.. (2022). "An exploratory study of information cocoon on short-form video platform", in: CIKM '22: Proceedings of the 31st ACM international conference on Information & Knowledge Management, 4178–4182.

[ref36] Li X. Liu Y. (2023). How does the converging media influence the political trust among Chinese youth? ——review based on chained dual mediation model. J. Mass Commun. Month. 9, 46–57. doi: 10.15897/j.cnki.cn51-1046/g2.2023.09.002

[ref37] Li C. Wen W. Liang H. (2013). “How personality influences users' needs for recommendation diversity?” in CHI '13 Extended Abstracts on Human Factors in Computing Systems, CHI EA '13, 829–834.

[ref38] Liu D. Ma Q. Yang H. (2008). Social cognition of migrant workers. Psychol. Sci. 31, 1373–1393. doi: 10.16719/j.cnki.1671-6981.2008.06.029

[ref39] Liu Y. Zhou S. (2019). Evolving Chinese nationalism: using the 2015 military parade as a case. East Asia 36, 255–270. doi: 10.1007/s12140-019-09314-w

[ref40] Lu Y. Chu Y. Shen F. (2016). Mass media, new technology, and ideology: an analysis of political trends in China. Glob. Media China 1, 70–101. doi: 10.1177/2059436416648799

[ref41] Ma L. (2015). Trends of Chinese social thoughts in the last forty years. Beijing: Oriental Press.

[ref42] Ma Y. (2015). “Online Chinese nationalism and its nationalist discourses” in Routledge handbook of Chinese media. eds. Rawnsley G. D. Ming-yeh T. R. (London: Routledge).

[ref43] Ma D. Huang M. (2023). Attitudes polarization and false consensus in online public opinion. Chin. J. J. Commun. 45, 47–73. doi: 10.13495/j.cnki.cjjc.2023.07.008

[ref44] Ma D. Lu Y. (2019). Information access, authoritarian personality, ideology and cyber nationalism: an analysis of the formation mechanism of Chinese Netizens' political attitudes. J. Tsinghua Univ. 34, 180–197. doi: 10.13613/j.cnki.qhdz.002856

[ref45] Ma D. Lu Y. (2022). Research on ideology of psychological orientation in foreign countries. Soc. Sci. For. Countr. 2, 152–200,

[ref46] Ma D. Lu M. (2023). The psychological root of public foreign policy inclination: a comparison of diplomatic belief systems between Chinese and American netizens. World Econ. Polit. 9, 144–172,

[ref47] Ma D. Wang L. (2015). An empirical analysis of Chinese Netizens' ideological positions and their formation. Society 35, 142–167. doi: 10.15992/j.cnki.31-1123/c.2015.05.007

[ref48] Ma D. Zhang Z. (2017). The homogeneity of public opinion and its psychological source: an empirical analysis based on a survey of Chinese netizens. J. Tsinghua Univ. 32, 174–195. doi: 10.13613/j.cnki.qhdz.002618

[ref49] Milad E. Bogg T. (2021). Spring 2020 COVID-19 surge: prospective relations between demographic factors, personality traits, social cognitions and guideline adherence, mask wearing, and symptoms in a U.S. Samples. Ann. Behav. Med. 55, 665–676. doi: 10.1093/abm/kaab039, PMID: 33991096 PMC8194716

[ref50] Mirels H. L. Dean J. B. (2006). Right-wing authoritarianism, attitude salience, and beliefs about matters of fact. Polit. Psychol. 27, 839–866. doi: 10.1111/j.1467-9221.2006.00540.x

[ref51] Nong L. Liao C. Ye J.-H. Wei C. Zhao C. Nong W. (2022). The STEAM learning performance and sustainable inquiry behavior of college students in China. Front. Psychol. 13:975515. doi: 10.3389/fpsyg.2022.975515, PMID: 36337577 PMC9631437

[ref52] Onraet E. Hiel A. V. Roets A. Cornelis I. (2011). The closed mind: “experience” and “cognition” aspects of openness to experience and need for closure as psychological bases for right-wing attitudes. Eur. J. Personal. 25, 184–197. doi: 10.1002/per.775

[ref53] Pan Y. Shu Z. (2023). Pro-liberalism vs. nationalism: how critical opinion leaders challenge the persuasive effect of propaganda in China. Chin. J. Commun., 1–17. doi: 10.1080/17544750.2023.2293860

[ref54] Pariser E. (2011). The filter bubble: What the internet is hiding from you. New York: Penguin Press.

[ref55] Pinto-Bustamante B. J. Riaño-Moreno J. C. Clavijo-Montoya H. A. Cárdenas-Galindo M. A. Campos-Figueredo W. D. (2023). Bioethics and artificial intelligence: between deliberation on values and rational choice theory. Front. Robot. AI 10:1140901. doi: 10.3389/frobt.2023.1140901, PMID: 37457388 PMC10338331

[ref56] Prior M. (2007). Post-broadcast democracy: How media choice increases inequality in political involvement and polarizes elections. New York: Cambridge University Press.

[ref57] Qin J. Du Q. Deng Y. Zhang B. Sun X. (2023). How does short video use generate political identity? Intermediate mechanisms with evidence from China’s small-town youth. Front. Psychol. 14:1107273. doi: 10.3389/fpsyg.2023.1107273, PMID: 36777217 PMC9911650

[ref58] Schoen H. Schumann S. (2007). Personality traits, partisan attitudes, and voting behavior evidence from Germany. Polit. Psychol. 28, 471–498. doi: 10.1111/j.1467-9221.2007.00582.x

[ref59] Smith A. D. (2010). Nationalism: Theory, ideology, history. Cambridge: Polity Press.

[ref60] Steele L. G. Lynch S. M. (2013). The pursuit of happiness in China: individualism, collectivism, and subjective well-being during China's economic and social transformation. Soc. Indic. Res. 114, 441–451. doi: 10.1007/s11205-012-0154-1, PMID: 24288434 PMC3839668

[ref61] Stroud N. J. (2010). Polarization and partisan selective exposure. J. Commun. 60, 556–576. doi: 10.1111/j.1460-2466.2010.01497.x

[ref62] Su R. Shen W. (2020). Is nationalism rising in times of the COVID-19 pandemic? Individual-level evidence from the United States. J. Chin. Polit. Sci. 26, 169–187. doi: 10.1007/s11366-020-09696-2, PMID: 32952388 PMC7486972

[ref63] Sun Y. Zhang H. (2021). What motivates people to pay for online sports streaming? An empirical evaluation of the revised technology acceptance model. Front. Psychol. 12:619314. doi: 10.3389/fpsyg.2021.619314, PMID: 34122216 PMC8194353

[ref64] Sunstein C. R. (2009). Republic. Com 2.0. New York: Princeton UP.

[ref65] Tabachnick B. G. Fidell L. S. (2007). Using multivariate statistics. Needham Heights, MA: Allyn and Bacon.

[ref66] Tan X. Dou X. Dong H. (2020). Current situation and upgrading countermeasures of People’s sense of gain from the perspective of social stratification. J. Guangxi Normal Univ. 56, 1–13. doi: 10.16088/j.issn.1001-6597.2020.05.001

[ref67] Tang W. (2008). Public opinion and civil Society in China. Guangzhou: Sun Yat-sen University Press.

[ref68] Völker B. (2023). Networks in lockdown: the consequences of COVID-19 for social relationships and feelings of loneliness. Soc. Networks 72, 1–2. doi: 10.1016/j.socnet.2022.08.001, PMID: 35968494 PMC9359936

[ref69] Wiard V. Lits B. Dufrasne M. (2022). “The spy who loved me”: a qualitative exploratory analysis of the relationship between youth and algorithms. Front. Commun. 7:778273. doi: 10.3389/fcomm.2022.778273

[ref70] Willnat L. Metzgar E. T. (2012). "American perceptions of China and Chinese: do the media matter?" In: The 65th annual meeting of the world Association for Public Opinion Research (Hong Kong).

[ref71] Xia J. Wu D. Zhong X. Nie X. (2013). Reliability and validity of Chinese big five personality questionnaire-brief version in the application of nurses. Chin. J. Health Psychol. 21, 1648–1687. doi: 10.13342/j.cnki.cjhp.2013.11.007

[ref72] Xu P. Ye Y. Zhang M. (2022). Exploring the effects of traditional media, social media, and foreign media on hierarchical levels of political trust in China. Glob. Media China 7, 357–377. doi: 10.1177/20594364221115270

[ref73] Yang S. Guo Y. Hu X. Shu S. Li J. (2016). Do lower class people have higher levels of system rationalization? -- A study from the perspective of social cognition. Acta Psychol. Sin. 48, 1467–1478. doi: 10.3724/SP.J.1041.2016.01467

[ref74] Yu G. Fang K. (2020). Does algorithmic content push lead to information cocoon? -- an empirical analysis of media diversity and source trust. Shandong Soc. Sci. 11, 170–169. doi: 10.14112/j.cnki.37-1053/c.2020.11.026

[ref75] Yu X. Wang J. (2023). Analysis of influencing factors of political support of Chinese youth: political concept, media use, social capital and intergenerational differences. Chin. Youth Soc. Sci. 42, 50–64. doi: 10.16034/j.cnki.10-1318/c.2023.02.016

[ref76] Yuan X. Wang C. (2022). Research on the formation mechanism of information cocoon and individual differences among researchers based on information ecology theory. Front. Psychol. 13:1055798. doi: 10.3389/fpsyg.2022.1055798, PMID: 36605281 PMC9809296

[ref77] Zhang D. (2020). China’s digital nationalism: search engines and online encyclopedias. J. Commun. Media Stud. 5, 1–19. doi: 10.18848/2470-9247/CGP/v05i02/1-19

[ref78] Zhang C. (2022). Contested disaster nationalism in the digital age: emotional registers and geopolitical imaginaries in COVID-19 narratives on Chinese social media. Rev. Int. Stud. 48, 219–242. doi: 10.1017/S0260210522000018

[ref79] Zhang D. Jamali A. B. (2022). China’s “weaponized” vaccine: intertwining between international and domestic politics. East Asia 39, 279–296. doi: 10.1007/s12140-021-09382-x, PMID: 35079216 PMC8776365

[ref80] Zhang D. Xu Y. (2023). When nationalism encounters the COVID-19 pandemic: understanding Chinese nationalism from media use and media trust. Glob. Soc. 37, 176–196. doi: 10.1080/13600826.2022.2098092

